# Supplementary material for: Balancing the efficacy and safety of chimeric antigen receptor T-cell therapy by affinity combination
Source: Nat Commun. 2026 Apr 10;17:3413. doi: 10.1038/s41467-026-71354-7 (PMC13069115; doi:10.1038/s41467-026-71354-7)
Supplement: Supplementary file 1 — Supplementary Information [file 41467_2026_71354_MOESM1_ESM.pdf]

# Supplementary file

# Containing supplementary figures 1-13

# Balancing the efficacy and safety of Chimeric Antigen Receptor T cell therapy by affinity combination


**AUTHORS**

Linda Warmuth[1,2,†], Sarah Dötsch[1,†], Manuel Trebo[1], Sarah Bellucci[1], Sophia Engels[1], Rafael Valdivia Manrique[1], Karl Moukarzel[1,2], Julius M. Schütz[1,2], Monika Hammel[1], Adrian Straub[1], Sabrina Wagner[1,2], Anna Hochholzer[1], Ciro Salinno[1], Jacqueline Seigner[3], Charlotte U. Zajc[3,4], Georg P. Schmidt[5], Josefine Michael[6], Thomas Nerreter[6], Michael Hudecek[6,7,8,9], Michael W. Traxlmayr[3], Monica Casucci[10], Stanley R. Riddell[11], Mateusz P. Poltorak[1], Dirk H. Busch[1,12,*,§] and Elvira D'Ippolito[1,12,*,§]

**AFFILIATIONS**

[1]Institute for Medical Microbiology, Immunology and Hygiene, Technical University of Munich, Munich, Germany.

[2]Graduate Center of Medicine and Health, TUM Graduate School, Technical University of Munich, Munich, Germany.

[3]Institute of Biochemistry, Department of Natural Science and Sustainable Resources, BOKU University, Vienna, Austria.

[4]Medical University of Vienna, Department of Transfusion Medicine and Cell Therapy, Vienna, Austria.

[5]Clinic and Polyclinic for Gynecology, TUM University Hospital, TUM School of Medicine, Technical University of Munich, Munich, Germany.

[6]Chair for Cellular Immunotherapy, Department of Medicine II, University Hospital Würzburg, Würzburg, Germany.

[7]Fraunhofer Institute for Cell Therapy and Immunology (IZI), Leipzig & Branch Site Cellular Immunotherapy, Würzburg, Germany.

[8]National Center for Tumor Diseases (NCT), Site Würzburg-Erlangen-Regensburg-Augsburg (WERA), Würzburg, Germany.

[9]Bavarian Center for Cancer Research (BZKF), Lighthouse Cellular Immunotherapies, Würzburg, Germany.

[10]Innovative Immunotherapies Unit, Division of Immunology, Transplantation, and Infectious Diseases, IRCCS Ospedale San Raffaele Scientific Institute, Milan, Italy.

[11]Translational Sciences and Therapeutics Division, Fred Hutchinson Cancer Center, Seattle, WA, USA.

[12]German Center for Infection Research (DZIF), partner site Munich, Germany.

[†] These authors contributed equally

[§] These authors jointly supervised this work

* Corresponding authors: elvira.dippolito@tum.de and dirk.busch@tum.de (+49-8941404120)

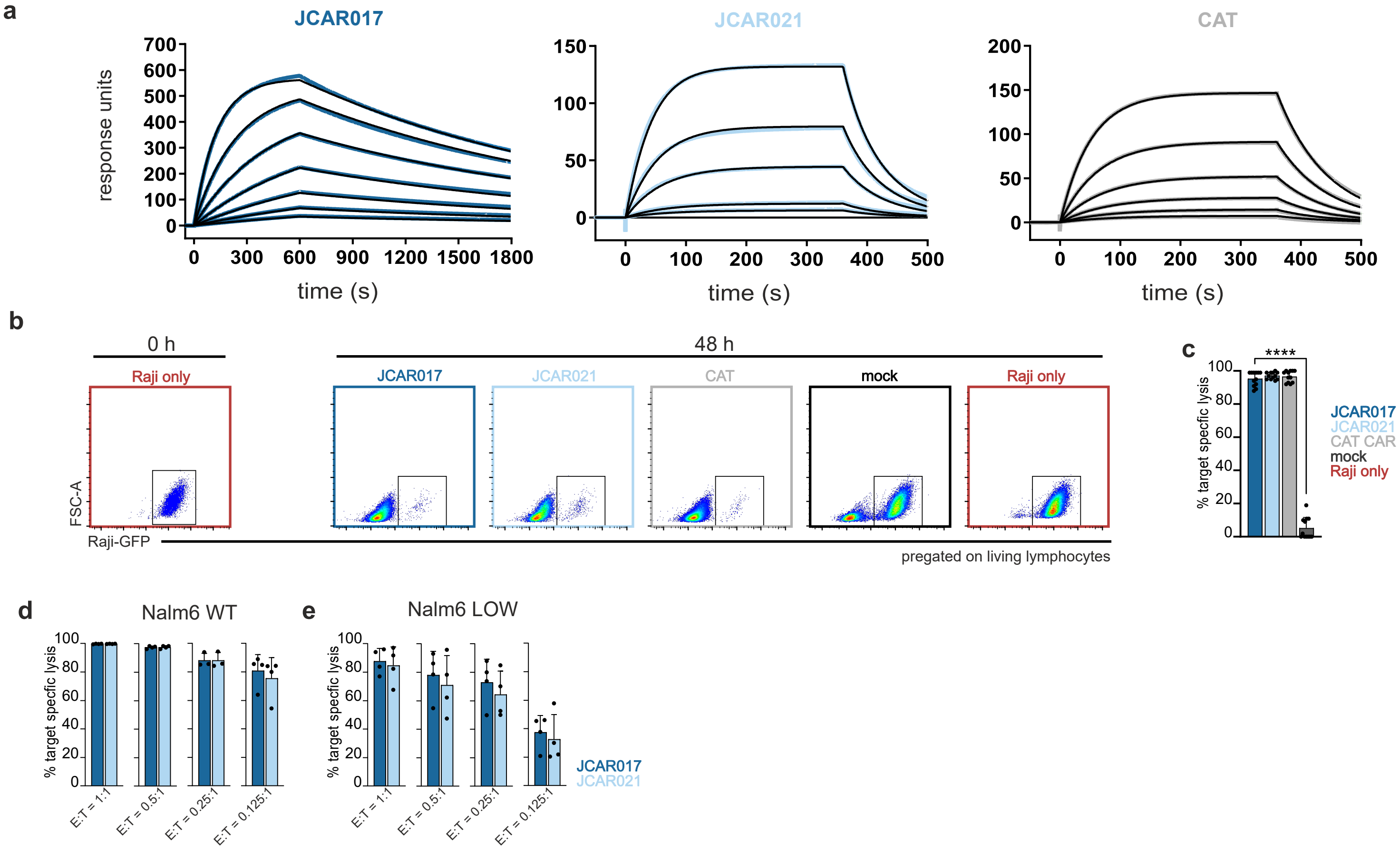


**Figure S1. Characterization of the functionality of CAR-T cells. Related to Figure 1. a)** SPR sensorgrams depicting the binding kinetics of JCAR017, JCAR021, and CAT CAR scFvs to varying concentrations of the soluble recombinant extracellular domain of CD19 (80, 40, 20, 10, 5, 2.5, 1.25, and 0.625 nM). **b**) Representative flow cytometry plots from cytotoxicity assay, illustrating CD19$^{+}$ GFP$^{+}$ Raji tumor cells at 0 and 48 h after co-culture with CAR-engineered primary human T cells. **c**) Quantification of target lysis following 48 h co-culture. Dots represent mean of technical triplicates. Data are displayed as mean + SD (n = 12). d-e) Quantification of target lysis following 24 h co-culture with wildtype d) or CD19-low-expressing e) Nalm-6 target cells at the indicated effector to target ratios. Dots represent the mean of technical triplicates. Data are displayed as mean + SD (n = 4). Statistical significance was determined by one-way ANOVA with JCAR017 as reference.

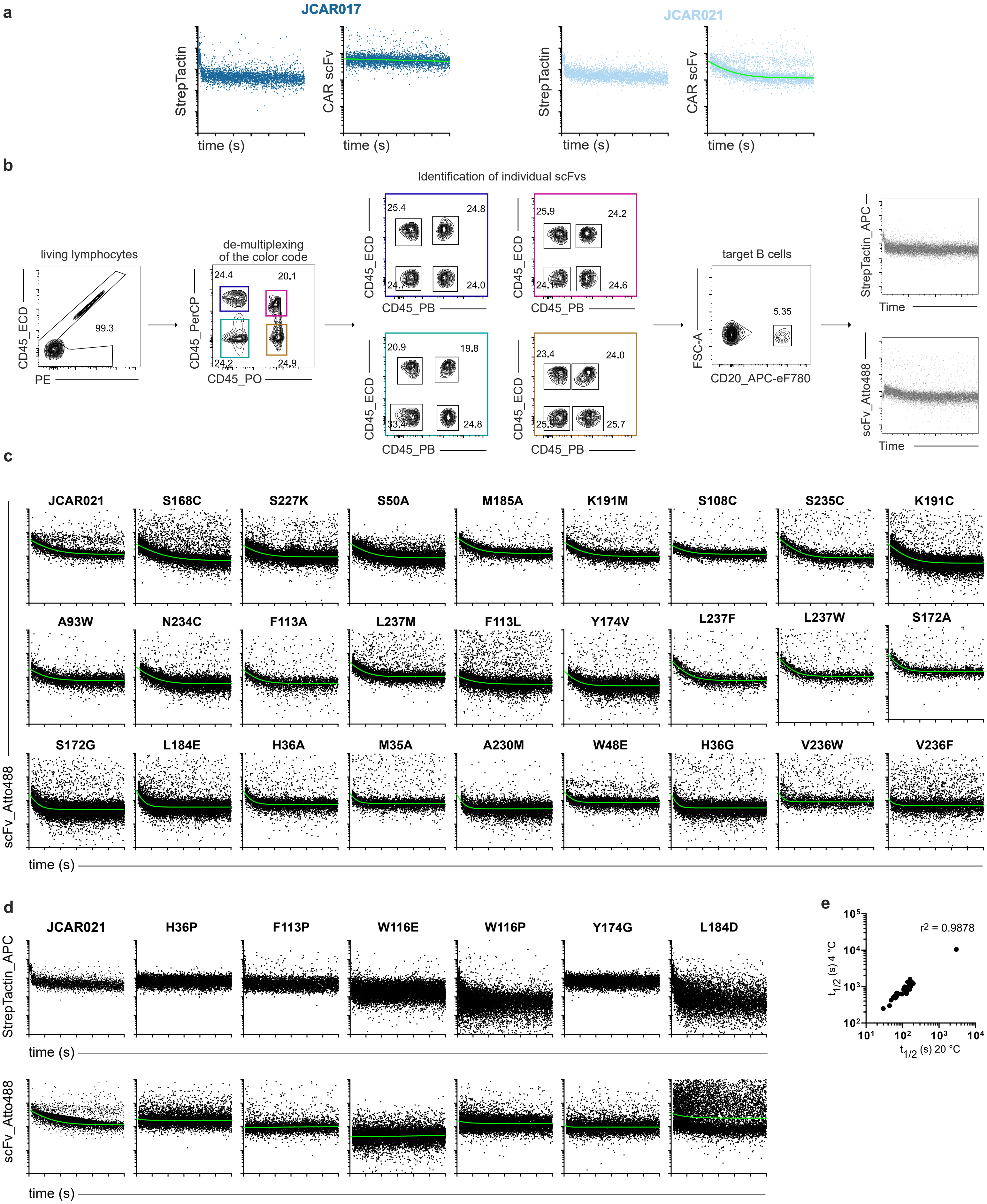


**Figure S2. Characterization of the binding properties of a scFv library. Related to Figure 2**. **a)** Representative flow cytometry plots showing the dissociation kinetics of the *Strep*Tactin-APC backbone and the Atto488-conjugated scFv (high-affinity JCAR017 and low-affinity JCAR021) following D-biotin addition (30 minutes, 20°C). The green line represents the fitted dissociation curve. **b)** Gating strategy for simultaneous $k_{off}$-rate measurement of 16 scFvs using unique combinations of anti-CD45 monoclonal antibody labels. **c)** Representative flow cytometry plots of robust and reproducible dissociation kinetics of Atto488-conjugated scFvs after D-biotin addition (30 minutes, 20°C). The green line indicates curve fitting. **d)** Representative flow cytometry plots showing dissociation of the backbone and scFv (30 minutes, 20°C) of clones with suboptimal *Strep*Tamer staining. The green line represents curve fitting. **e)** Pearson correlation of flow $k_{off}$-rate measurements performed at 4 °C or 20 °C.

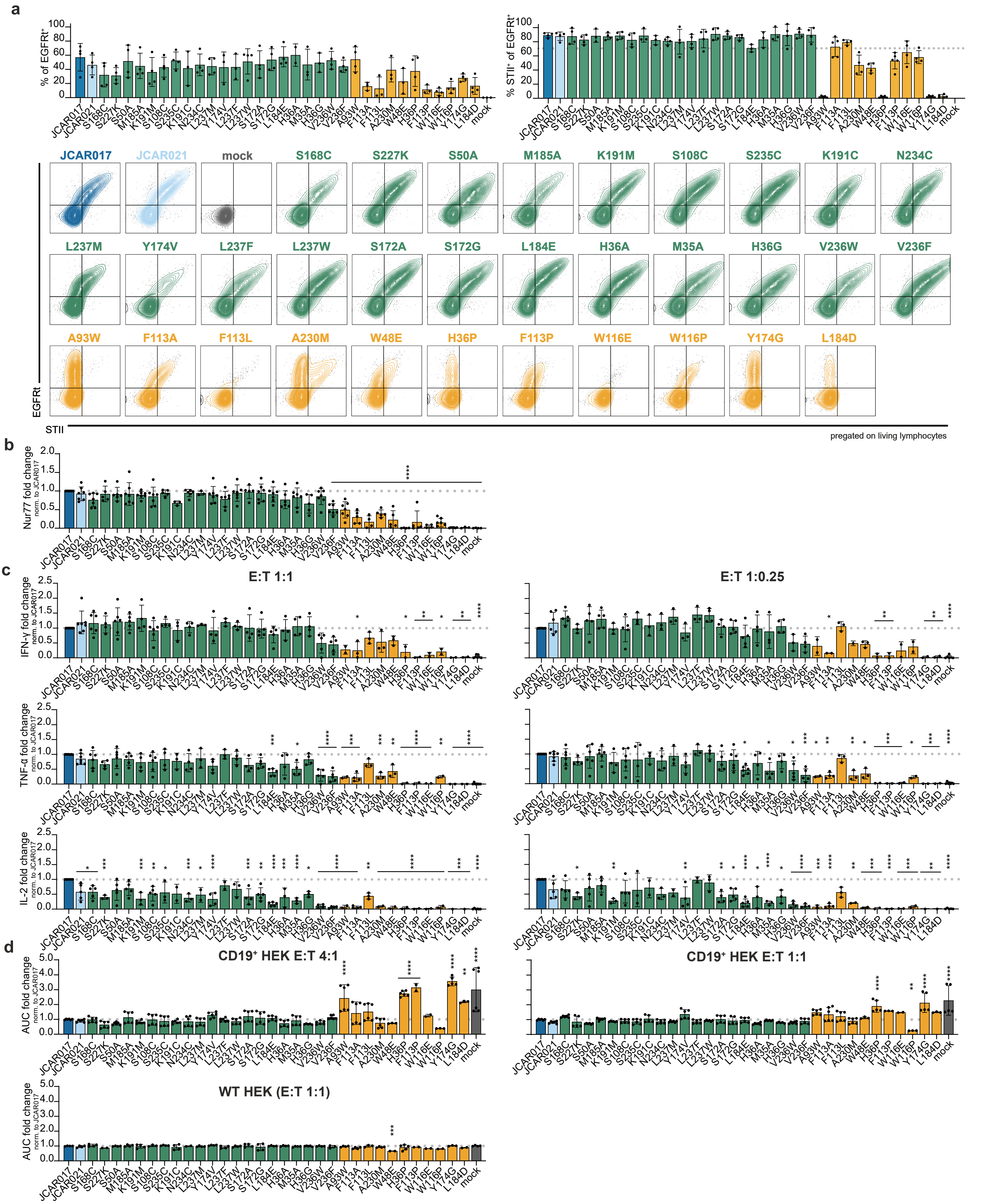


**Figure S3. In vitro functionality is maintained over a large breadth of affinity. Related to Figure 2. a**) Transduction efficiency (EGFRt⁺) and CAR surface expression (STII⁺) among transduced primary human T cells. Representative flow cytometry plots of the JCAR021 mutant library are shown below (n = 3-4). **b**) Nur77-tdTomato expression levels in Jurkat cells engineered with the indicated CAR construct following co-culture with CD19⁺ GFP⁺ Raji tumor cells (E:T 1:1) (n = 3-5). **c**) Intracellular cytokine production of IFN-γ, TNF- and IL-2 in primary human T cells after 5 h co-culture with Raji tumor cells at E:T ratios of 1:1 and 1:0.25 (n = 2-3). **d**) Impedance-based xCelligence measurements, quantified as area under the curve (AUC) following co-culture with CD19⁺ or WT HEK cells at E:T ratios of 4:1 and 1:1 (n = 2-3). Data are displayed as mean ± SD. Each dot represents the mean of technical replicates of each independent experiment. In (b-d), data were normalized to JCAR017, and dotted gray lines indicate the JCAR17 reference value. Statistical analysis was performed using one-way ANOVA for multiple comparisons, with JCAR017 as reference. *p < 0.05, **p < 0.01, ***p ≤ 0.001, ****p ≤ 0.0001.

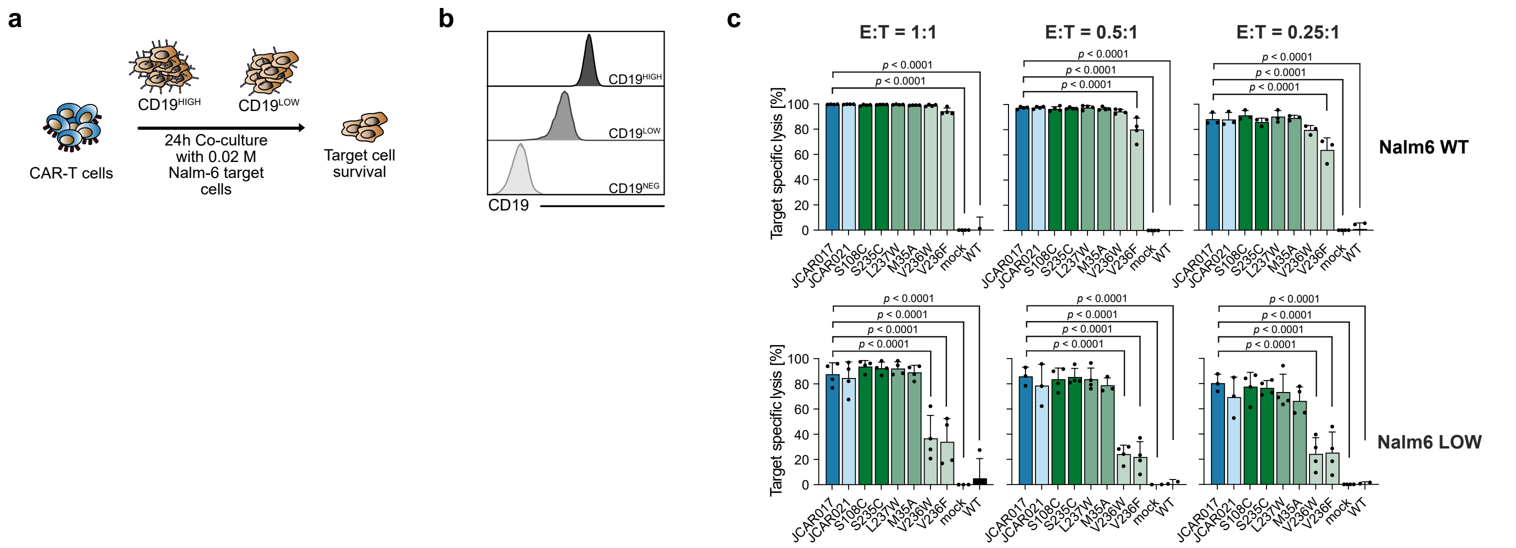


**Figure S4. In vitro cytotoxicity across varying effector-to-target ratios and target antigen densities. Related to figure 2.** **a**) Overview of the experimental setup. $2x10^5$ Nalm6-ffluc-GFP cells, either wild-type or CD19-low, were co-cultured with the indicated doses of CAR-T cells for 24 h prior to acquisition. **b**) Flow cytometric evaluation of CD19 antigen expression levels in the different Nalm6 models. **c**) Quantification of cell killing. Each dot represents the mean of three technical replicates of an independent biological replicate (n = 4), and data are shown as mean + SD. Statistical analyses were performed using one-way ANOVA for multiple comparisons, with JCAR017 as the reference.

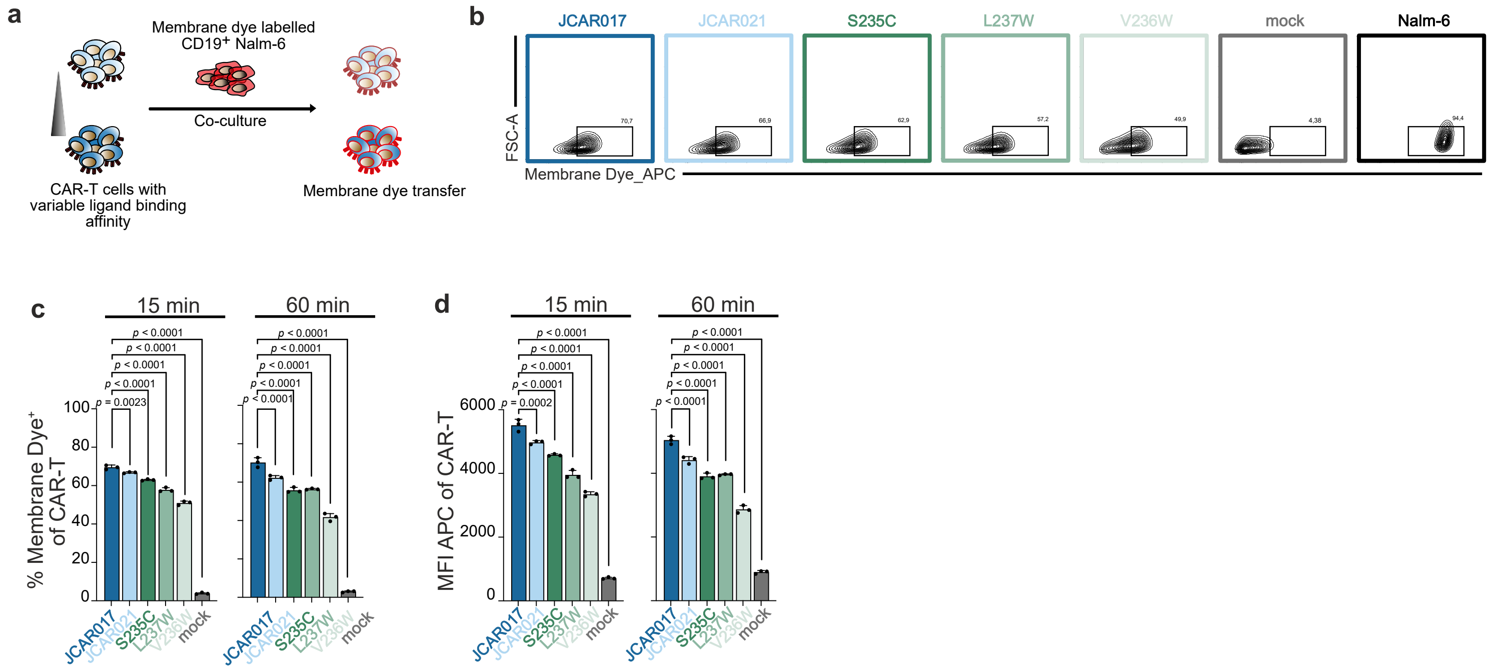


**Figure S5. The extent of target membrane interaction decreases with lower CAR ligand binding affinity. Related to Figure 2. a)** Overview of the experimental setup to investigate the interaction between CAR-T cells and their target cells' membrane. **b)** Representative flow cytometry plots showing membrane interaction of JCAR017, JCAR021 and selected mutants with dye-labeled CD19$^+$ Nalm-6 cells after co-culture at a 1:1 E:T for 15 min. **c)** Frequencies of membrane dye$^+$ CAR-T cells and **d)** mean fluorescence intensity (MFI) of APC-membrane dye of CAR-T cells following 15 and 60 min co-culture with membrane dye-labeled target cells. Data are one representative of two biological replicates with technical triplicates (n = 2). Data are shown as mean + SD. Statistical analysis was performed using one-way ANOVA for multiple comparisons with JCAR017 as reference.

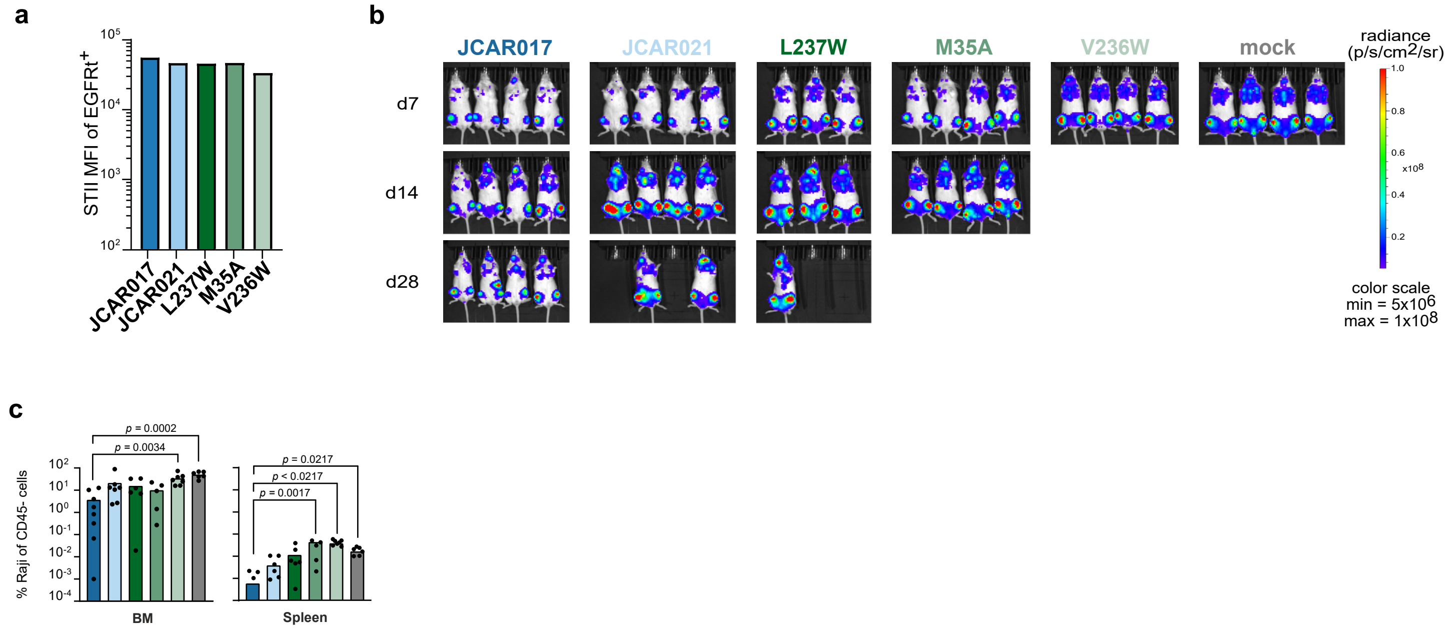


**Figure S6. Reduced CAR ligand binding affinity heavily influences *in vivo* anti-tumor efficacy. Related to Figure 3. a)** STII mean fluorescent intensities (MFI) of EGFRt⁺ CAR-transduced T cells **b)** Representative bioluminescence images showing Raji tumor growth in mice treated with different CAR-T cell products. **c)** Frequencies of GFP⁺ Raji tumor cells in the bone marrow and spleen of treated mice (n = 5-8). Statistical analysis was performed using one-way ANOVA for multiple comparisons with JCAR017 as reference.

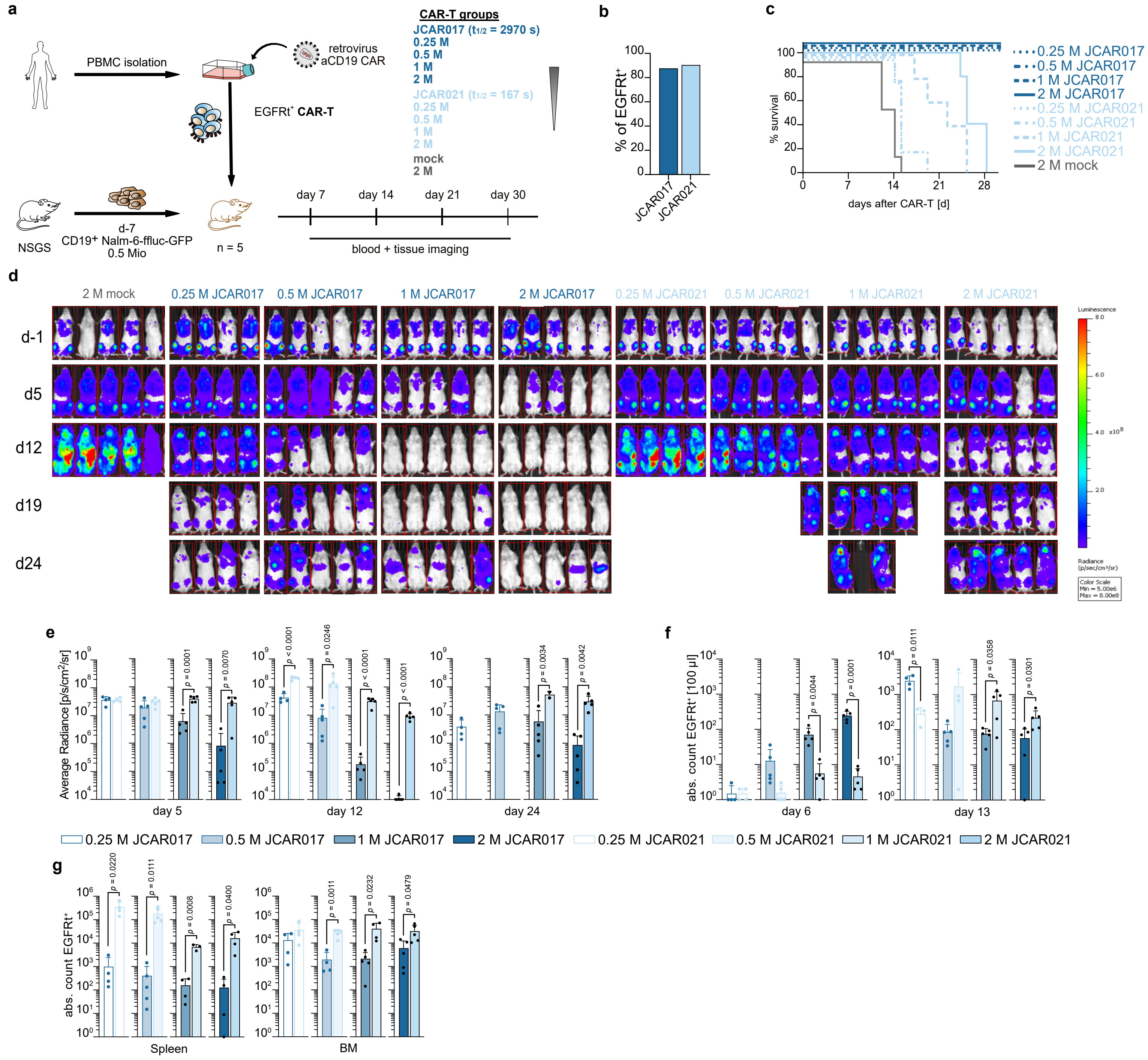


**Figure S7. In vivo functionality of high- and low-affinity CAR-T cells in a xenograft model. Related to Figure 3. a)** Schematic depicting experimental setup employing bulk CAR-T cells. **b)** Transduction efficiency of infused CAR-T cell products. **c)** Kaplan-Meier survival curve of mice treated with escalating JCAR017 and JCAR021 CAR-T cell doses. **d)** Bioluminescence images showing residual Nalm-6 tumor burden post treatment. **e)** Quantification of tumor burden (photons/second/cm²/steradian) post CAR-T cell injection. **f)** Absolute CAR-T cell counts in 100 µl blood at the indicated time post treatment. **g)** Absolute CAR-T cell levels in spleen and BM at sacrifice. In (e-g) data are shown as mean + SD. Each dot represents an individual mouse. Statistical analysis was performed using a two-tailed unpaired t-test comparing individual CAR-T cell doses.

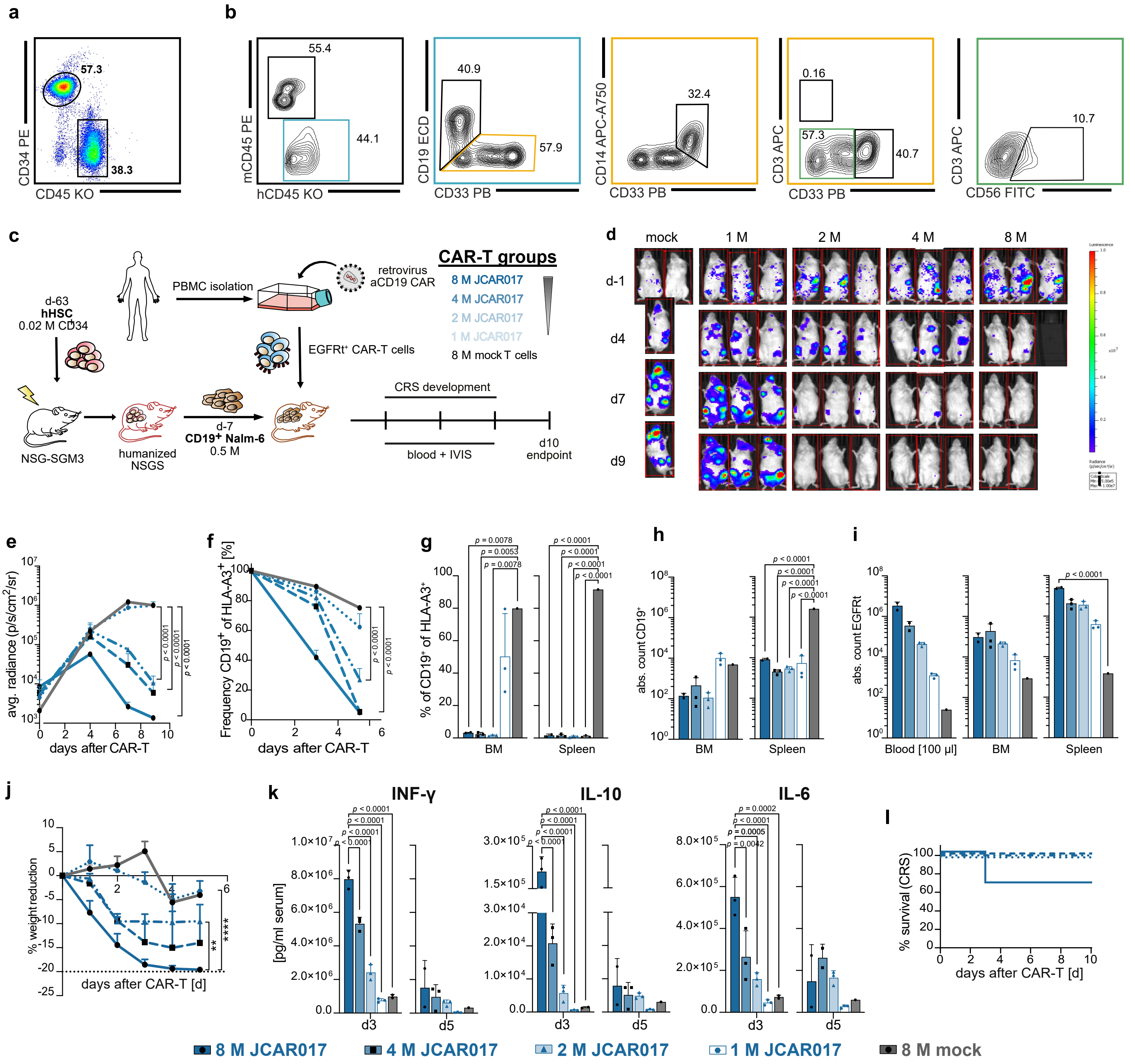


**Figure S8. Toxicity of high-affinity CAR-T cells in a humanized mouse model. Related to Figure 4. a**) Flow cytometry dot plot of purified human hematopoietic stem cells (HSCs) prior to injection into NSG-S mice. **b**) Gating strategy used to determine the frequency of humanization and immune compartment reconstitution. **c**) Schematic overview of the experimental design for dose titration of high-affinity JCAR017 CAR-T cells in a humanized CRS model using Nalm-6 ffluc tumor cells and sorted CAR-T cells. **d**) Bioluminescence images showing Nalm-6 tumor growth in mice treated with varying CAR-T cell doses. **e**) Residual tumor burden of Nalm-6 cells assessed by bioluminescence imaging, quantified as the maximum photons per second per cm2 per steradian. **f**) Kinetics of CD19⁺ B cells in the blood depicted as frequencies. **g**) Frequencies and **h**) absolute cell count of CD19⁺ B cells in the bone marrow and spleen. **i**) Absolute CAR-T cell counts in 100 µL of blood, whole bone marrow, and spleen at the time of sacrifice. **j**) Depiction of weight developments during the acute phase of CRS (five days), normalized to the starting weight before CAR-T cell transfer. **k**) Serum cytokine concentration of IFN-γ, IL-10 and IL-6 three- and five-days post-transfer. i) Kaplan-Meier survival curve depicting CRS-related mortality following CAR-T cell transfer. Data are shown as mean + SD (n = 3). Statistical analysis was performed using two-way ANOVA (e, f and j) or one-way ANOVA (g, h, i and k) for multiple comparison with mock as reference.

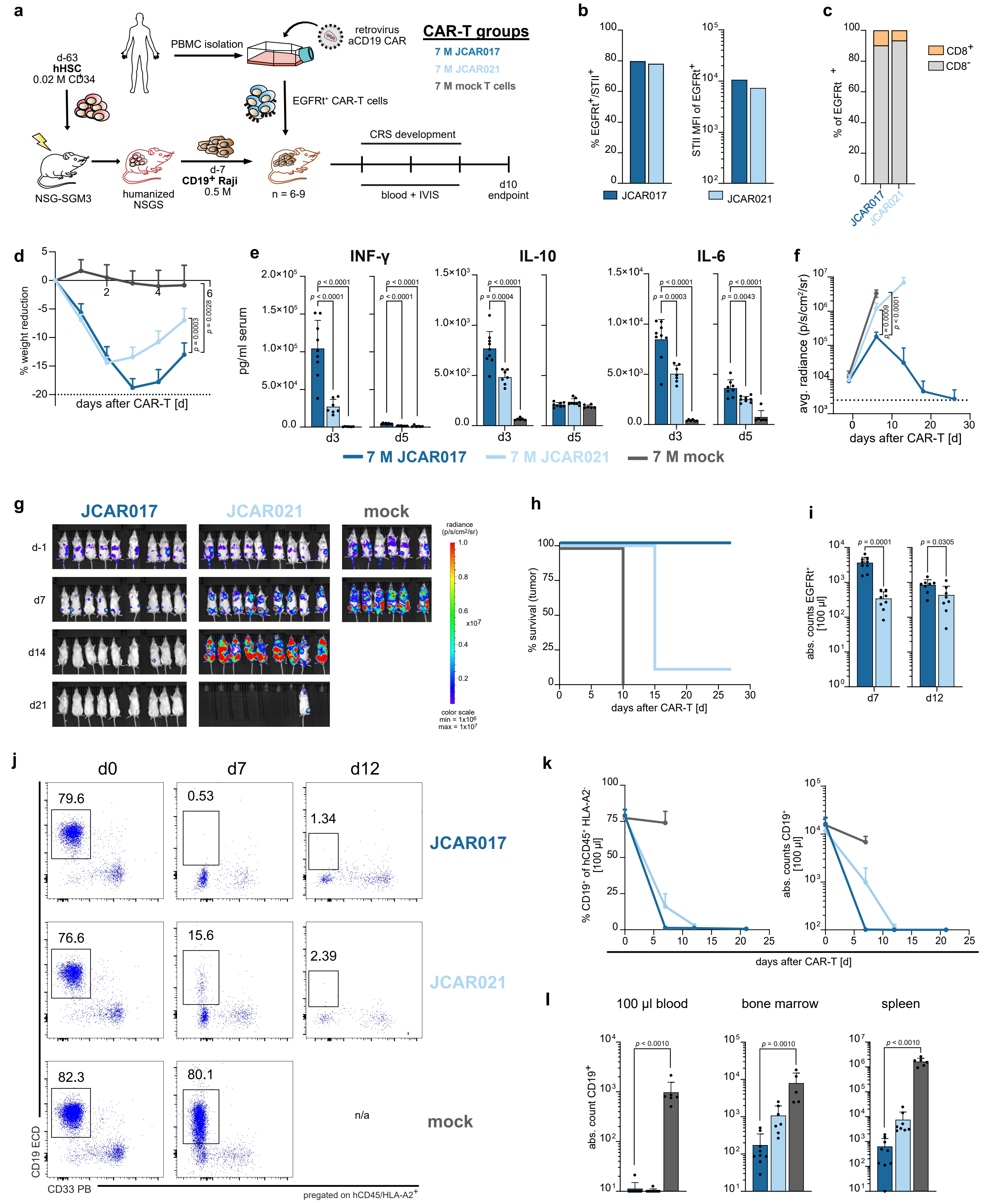


**Figure S9. Toxicity of high- and low-affinity CAR-T cells in a humanized mouse model. Related to Figure 4. a**) Schematic overview of the experimental design for comparing high- and low-affinity CAR-T cells in a humanized CRS model using Raji-6 ffluc tumor cells. **b**) Transduction efficiencies of JCAR017 and JCAR021 represented by the frequencies of EGFRt+/STII+ cells (left) and STII mean fluorescent intensity (MFI) within EGFRt+ T cells (right). **c**) Distribution of CD8+ to CD8- population among CAR-transduced T cells in the infusion product. **d**) Kinetics of weight during the first five days post CAR-T cell transfer. **e**) Serum cytokine levels (IFN-γ, IL-10 and IL-6) three and five days after CAR-T cell transfer. **f**) Residual tumor burden quantified via bioluminescence imaging (maximum photon/second/cm$^2$/ steradian). **g**) Bioluminescence images showing residual Raji tumor burden in mice following treatment. **h**) Kaplan-Meier survival curve for mice treated with JCAR017- and JCAR021-transduced T cells. **i**) Absolute CAR-T cell counts in 100 µl blood measured seven- and 12-days post-transfer. **j-l**) Flow cytometry plots, kinetics and absolute counts of CD19+ B cells in peripheral blood, bone marrow and spleen at the time of sacrifice. Data are shown as mean + SD (n = 6-9). Statistical analysis was performed using two-way ANOVA (d and f), one-way ANOVA (e and l) for multiple comparisons, with JCAR017 as reference group and an unpaired, two-tailed t-test (i).

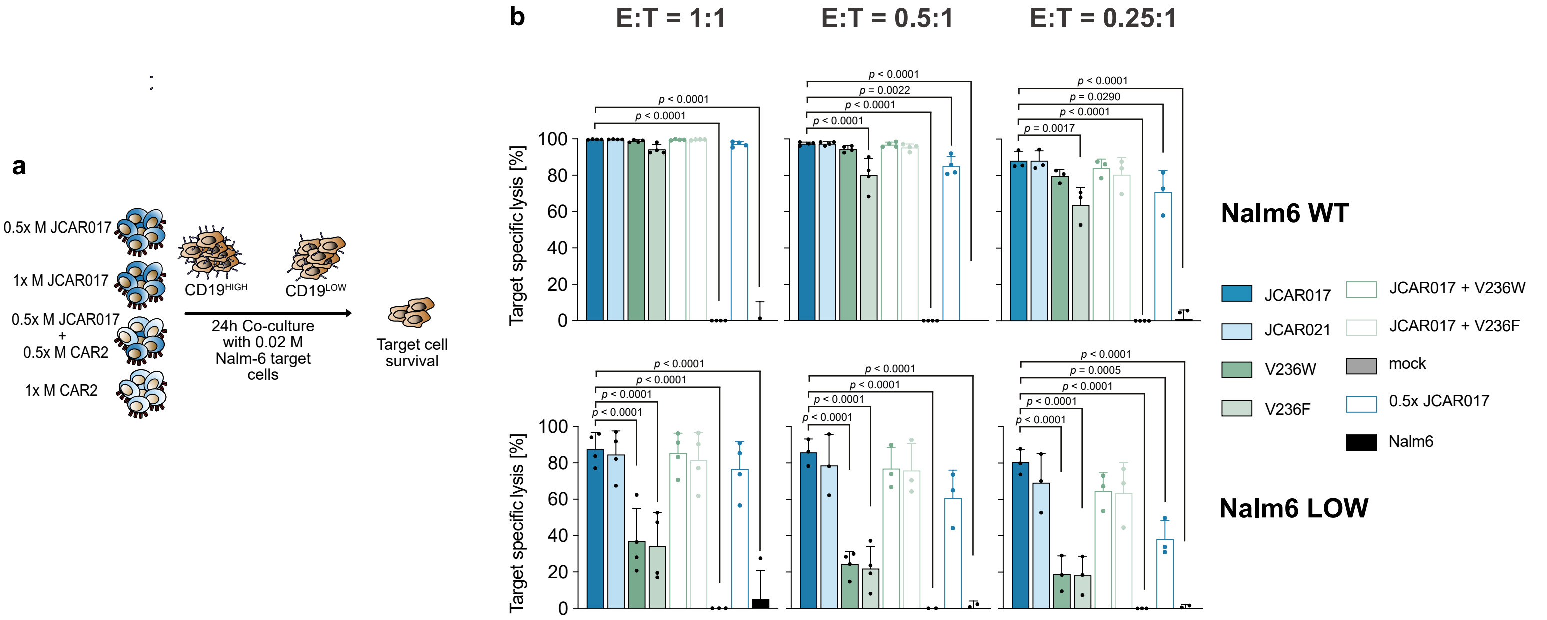


**Figure S10.** ***In vitro*** **cytotoxicity of CAR-T cell products combining high- and low-affinity receptors. Related to Fig.5. a)** Schematic depiction of the experimental setup. 2x10⁵ Nalm6-ffluc-GFP cells, either wild-type or CD19-low, were co-cultured with the indicated doses of CAR-T cells for 24 h prior to acquisition **b**) Quantification of killing capacity. Statistical analysis was performed using one-way ANOVA for multiple comparisons, with JCAR017 as reference group.

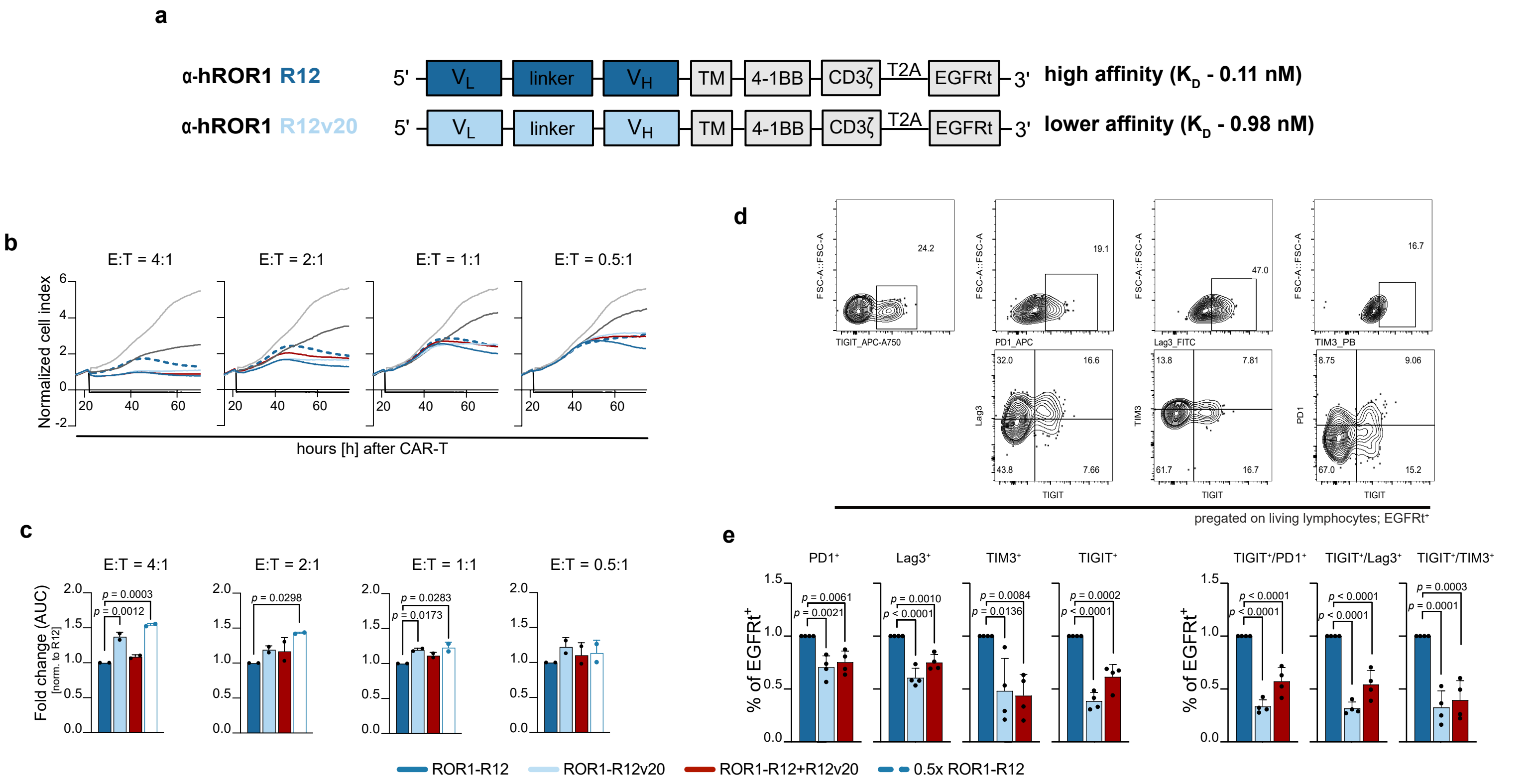


**Figure S11. Cytotoxicity and exhaustion profiles of CAR-T cell products combining high- and low-affinity ROR1-specific receptors. Related to Fig.5. a**) Schematic of CAR construct structures for lentiviral gene delivery. **b**) Representative xCelligence impedance-based killing curves at the indicated effector-to-target (E:T) ratios. **c**) Quantification of impedance-based cytotoxicity by the area under the curve (AUC). **d**) Gating strategy to evaluate exhaustion profile of affinity combined CAR-T cells. **e**) Expression (left) and co-expression (right) of exhaustion markers following 14 days of chronic antigen exposure. Each dot represents the mean of technical triplicates of an independent biological experiment (n = 2-4). Data are normalized to the high-affinity CAR R12. Statistical analyses were performed using one-way ANOVA for multiple comparisons with the high-affinity CAR-T cell condition (R12) as reference.

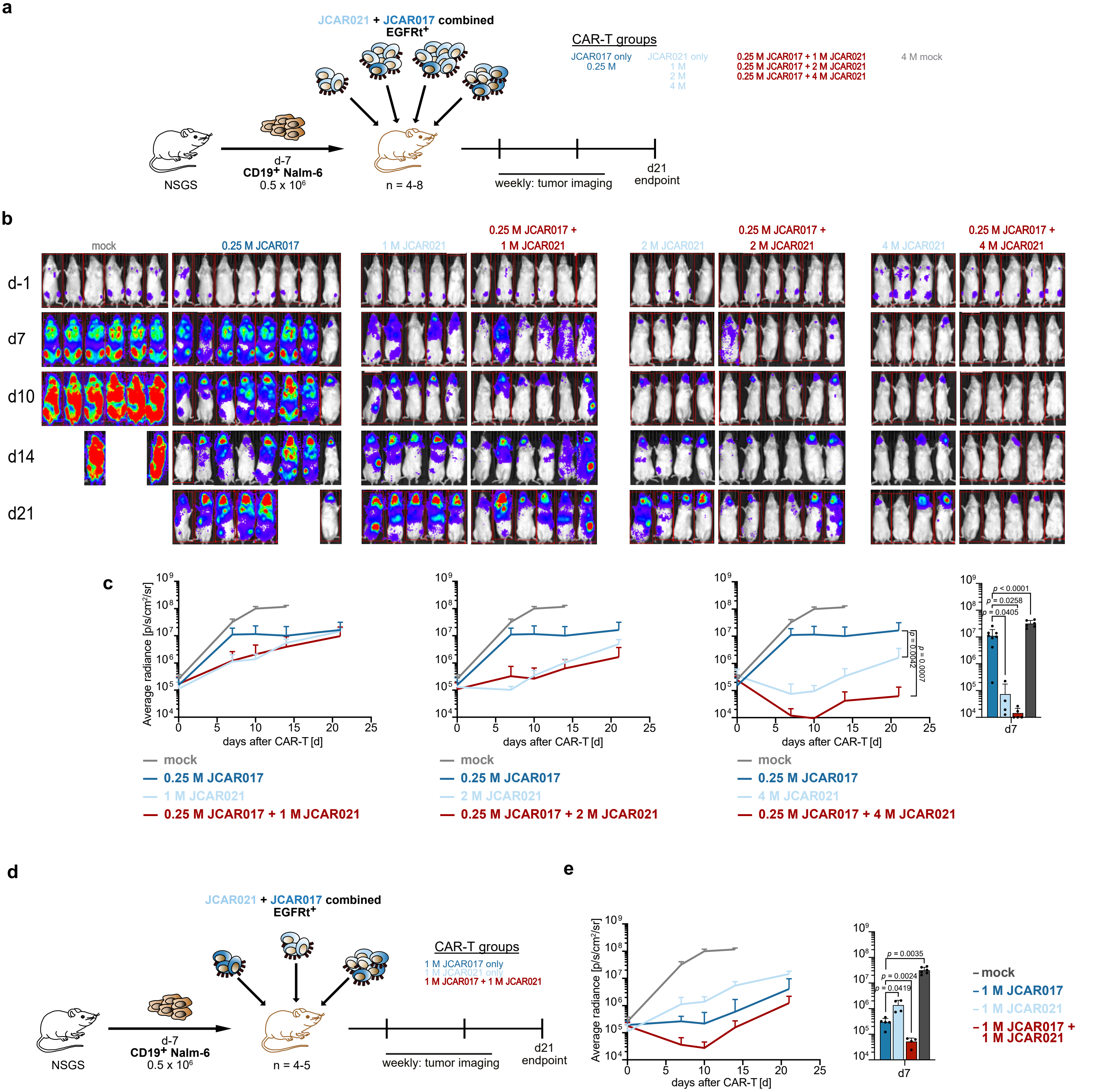


**Figure S12. Anti-tumor efficacy of an affinity-combined CAR-T cell product in a xenograft model. Related to Figure 6. a)** Schematic representation of the experimental design. **b)** Representative bioluminescence images of tumor-bearing NSG-S mice treated with CAR-T cells. **c)** Quantification of tumor burden measured by bioluminescence signal as the maximum photons per second per cm$^2$ per steradian. Data are represented as mean + SD of bioluminescence signal (n = 4-8). **d)** Schematic representation of the experimental design **e)** Quantification of residual tumor burden at seven days post-transfer (middle) and during the experimental course (right). Data are expressed as mean + SD of bioluminescence signal (n = 5-6). Statistical analysis was performed using one-way ANOVA (bar graphs in c and e) or two-way ANOVA (c and e) for multiple comparisons, with JCAR017 as reference group.

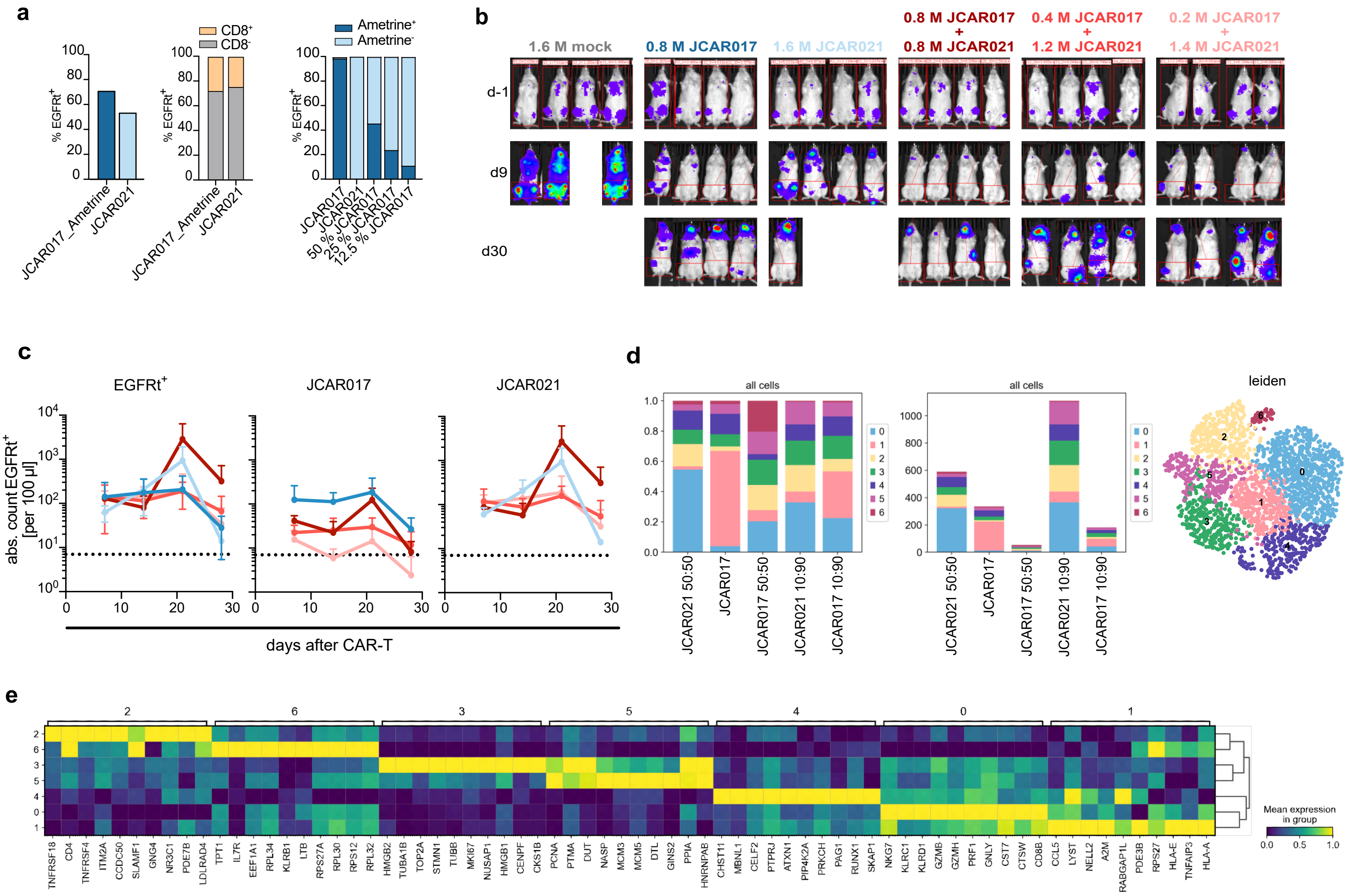


**Figure S13. Affinity combination improves functionality of high-affinity CAR-T cells. Related to Figure 6. a)** Evaluation of transduction efficiency (left), CD8⁺ to CD8⁻ distribution within CAR-transduced EGFRt⁺ T cells (middle) and CAR composition based on co-expression of Ametrine within EGFRt⁺ T cells. **b)** Representative bioluminescence images of tumor-bearing NSG-S mice treated with CAR-T cell products combining varying affinities. **c)** Quantification of EGFRt⁺ cell counts in 100 µL blood (left), along with the proportions of Ametrine⁺ (JCAR017) (middle) and Ametrine⁻ (JCAR021) (right) cells within the EGFRt⁺ population. **d)** Distribution of percentages (left) and absolute cell counts (right) of cells isolated from specific mice, attributed to high-affinity JCAR017 or low-affinity JCAR021 defining distinct clusters (0-6). **e)** Top ten differentially expressed genes characterizing the individual clusters defined in (d).
