# Supplementary material for: Balancing the efficacy and safety of chimeric antigen receptor T-cell therapy by affinity combination
Source: Nat Commun. 2026 Apr 10;17:3413. doi: 10.1038/s41467-026-71354-7 (PMC13069115; doi:10.1038/s41467-026-71354-7)
Supplement: Supplementary file 2 — Reporting Summary [file 41467_2026_71354_MOESM2_ESM.pdf]

nature portfolio

Corresponding author(s): Dirk H. Busch; Elvira D'Ippolito

Last updated by author(s): 2026 Feb 15

Reporting Summary

Nature Portfolio wishes to improve the reproducibility of the work that we publish. This form provides structure for consistency and transparency in reporting. For further information on Nature Portfolio policies, see our Editorial Policies and the Editorial Policy Checklist.

Statistics

For all statistical analyses, confirm that the following items are present in the figure legend, table legend, main text, or Methods section.

n/a	Confirmed	
☐	☒	The exact sample size (*n*) for each experimental group/condition, given as a discrete number and unit of measurement
☐	☒	A statement on whether measurements were taken from distinct samples or whether the same sample was measured repeatedly
☐	☒	The statistical test(s) used AND whether they are one- or two-sided<br>*Only common tests should be described solely by name; describe more complex techniques in the Methods section.*
☐	☒	A description of all covariates tested
☐	☒	A description of any assumptions or corrections, such as tests of normality and adjustment for multiple comparisons
☐	☒	A full description of the statistical parameters including central tendency (e.g. means) or other basic estimates (e.g. regression coefficient) AND variation (e.g. standard deviation) or associated estimates of uncertainty (e.g. confidence intervals)
☐	☒	For null hypothesis testing, the test statistic (e.g. F, t, r) with confidence intervals, effect sizes, degrees of freedom and P value noted<br>*Give P values as exact values whenever suitable.*
☒	☐	For Bayesian analysis, information on the choice of priors and Markov chain Monte Carlo settings
☐	☒	For hierarchical and complex designs, identification of the appropriate level for tests and full reporting of outcomes
☐	☒	Estimates of effect sizes (e.g. Cohen's *d*, Pearson's *r*), indicating how they were calculated

Our web collection on statistics for biologists contains articles on many of the points above.

Software and code

Policy information about availability of computer code

Data collection	CytExpert, FlowJo v10.8.0
Data analysis	GraphPad Prism Software 10.4.2, CellRanger 7.0.0. (10x Genomics) (https://doi.org/10.15252/msb.20188746 and https://www.scbestpractices.org/preamble.html). Data analysis was performed with SCANPY V1.9.1. and diffxpy (https://diffxpy.readthedocs.io/en/latest/index.html), GO analysis by g:Profiler toolkit (https://doi.org/10.1093/nar/gkad347) , GSEA by GSEAPY (https://doi.org/10.1093/bioinformatics/btac757

For manuscripts utilizing custom algorithms or software that are central to the research but not yet described in published literature, software must be made available to editors and reviewers. We strongly encourage code deposition in a community repository (e.g. GitHub). See the Nature Portfolio guidelines for submitting code & software for further information.

Data

Policy information about availability of data

All manuscripts must include a data availability statement. This statement should provide the following information, where applicable:

- Accession codes, unique identifiers, or web links for publicly available datasets
- A description of any restrictions on data availability
- For clinical datasets or third party data, please ensure that the statement adheres to our policy

The single cell RNA sequencing data generated in this study have been deposited in Gene Expression Omnibus under accession number GSE322706 (https://www.ncbi.nlm.nih.gov/geo/query/acc.cgi?acc=GSE322706). Processed single-cell datasets and all source data supporting the findings of this study have been deposited in the zenodo repository (DOI: 10.5281/zenodo.18861476). Source data are provided with this paper.

Research involving human participants, their data, or biological material

Policy information about studies with human participants or human data. See also policy information about sex, gender (identity/presentation), and sexual orientation and race, ethnicity and racism.

Reporting on sex and gender	PBMCs were obtained from the Blood donor service of Bavarian Red Cross Munich. Written informed consent was obtained from the participants and usage of the blood samples was approved according to national law by the local Institutional Review Board (Ethikkommission der Medizinischen Fakultät der Technischen Universität München). Sex was not a factor of choosing samples for this study.
Reporting on race, ethnicity, or other socially relevant groupings	Race and ethnicity were not factors of choosing samples for this study.
Population characteristics	For PBMCs from Blood donor service of Bavarian Red Cross Munich, population characteristics are reflective of healthy volunteers defined.
Recruitment	N/A
Ethics oversight	Ethikkommission der Medizinischen Fakultät der Technischen Universität München

Note that full information on the approval of the study protocol must also be provided in the manuscript.

Field-specific reporting

Please select the one below that is the best fit for your research. If you are not sure, read the appropriate sections before making your selection.

☒ Life sciences ☐ Behavioural & social sciences ☐ Ecological, evolutionary & environmental sciences

For a reference copy of the document with all sections, see nature.com/documents/nr-reporting-summary-flat.pdf

Life sciences study design

All studies must disclose on these points even when the disclosure is negative.

Sample size	The in vitro experiments were performed with n = 3-6 donors per group and are statistically rigorous to detect the difference based on our experiences and prior data. Sample size for mouse experiments were calculated by a professional biostatistician and is based on exploratory orientation studies and was approved by the government of upper bavaria Department 5 - Environment, Health and Consumer Protection. The details of samples and statistics have been described in the figure legends.
Data exclusions	No data were excluded from analysis in each experiment
Replication	all attempts at replication were successful in each experiment
Randomization	Mice were randomly allocated to experimental groups
Blinding	Experimenters responsible for infusions, sample collection, preparation and flow cytometry analysis were blinded to the experimental group assignment

Behavioural & social sciences study design

All studies must disclose on these points even when the disclosure is negative.

Study description	*Briefly describe the study type including whether data are quantitative, qualitative, or mixed-methods (e.g. qualitative cross-sectional, quantitative experimental, mixed-methods case study).*
Research sample	*State the research sample (e.g. Harvard university undergraduates, villagers in rural India) and provide relevant demographic information (e.g. age, sex) and indicate whether the sample is representative. Provide a rationale for the study sample chosen. For studies involving existing datasets, please describe the dataset and source.*
Sampling strategy	*Describe the sampling procedure (e.g. random, snowball, stratified, convenience). Describe the statistical methods that were used to predetermine sample size OR if no sample-size calculation was performed, describe how sample sizes were chosen and provide a rationale for why these sample sizes are sufficient. For qualitative data, please indicate whether data saturation was considered, and what criteria were used to decide that no further sampling was needed.*
Data collection	*Provide details about the data collection procedure, including the instruments or devices used to record the data (e.g. pen and paper, computer, eye tracker, video or audio equipment) whether anyone was present besides the participant(s) and the researcher, and whether the researcher was blind to experimental condition and/or the study hypothesis during data collection.*
Timing	*Indicate the start and stop dates of data collection. If there is a gap between collection periods, state the dates for each sample cohort.*
Data exclusions	*If no data were excluded from the analyses, state so OR if data were excluded, provide the exact number of exclusions and the rationale behind them, indicating whether exclusion criteria were pre-established.*
Non-participation	*State how many participants dropped out/declined participation and the reason(s) given OR provide response rate OR state that no participants dropped out/declined participation.*
Randomization	*If participants were not allocated into experimental groups, state so OR describe how participants were allocated to groups, and if allocation was not random, describe how covariates were controlled.*

Ecological, evolutionary & environmental sciences study design

All studies must disclose on these points even when the disclosure is negative.

Study description	*Briefly describe the study. For quantitative data include treatment factors and interactions, design structure (e.g. factorial, nested, hierarchical), nature and number of experimental units and replicates.*
Research sample	*Describe the research sample (e.g. a group of tagged Passer domesticus, all Stenocereus thurberi within Organ Pipe Cactus National Monument), and provide a rationale for the sample choice. When relevant, describe the organism taxa, source, sex, age range and any manipulations. State what population the sample is meant to represent when applicable. For studies involving existing datasets, describe the data and its source.*
Sampling strategy	*Note the sampling procedure. Describe the statistical methods that were used to predetermine sample size OR if no sample-size calculation was performed, describe how sample sizes were chosen and provide a rationale for why these sample sizes are sufficient.*
Data collection	*Describe the data collection procedure, including who recorded the data and how.*
Timing and spatial scale	*Indicate the start and stop dates of data collection, noting the frequency and periodicity of sampling and providing a rationale for these choices. If there is a gap between collection periods, state the dates for each sample cohort. Specify the spatial scale from which the data are taken*
Data exclusions	*If no data were excluded from the analyses, state so OR if data were excluded, describe the exclusions and the rationale behind them, indicating whether exclusion criteria were pre-established.*
Reproducibility	*Describe the measures taken to verify the reproducibility of experimental findings. For each experiment, note whether any attempts to repeat the experiment failed OR state that all attempts to repeat the experiment were successful.*
Randomization	*Describe how samples/organisms/participants were allocated into groups. If allocation was not random, describe how covariates were controlled. If this is not relevant to your study, explain why.*
Blinding	*Describe the extent of blinding used during data acquisition and analysis. If blinding was not possible, describe why OR explain why blinding was not relevant to your study.*

Did the study involve field work? ☐ Yes ☐ No

Field work, collection and transport

Field conditions	*Describe the study conditions for field work, providing relevant parameters (e.g. temperature, rainfall).*
Location	*State the location of the sampling or experiment, providing relevant parameters (e.g. latitude and longitude, elevation, water depth).*
Access & import/export	*Describe the efforts you have made to access habitats and to collect and import/export your samples in a responsible manner and in compliance with local, national and international laws, noting any permits that were obtained (give the name of the issuing authority, the date of issue, and any identifying information).*
Disturbance	*Describe any disturbance caused by the study and how it was minimized.*

Reporting for specific materials, systems and methods

We require information from authors about some types of materials, experimental systems and methods used in many studies. Here, indicate whether each material, system or method listed is relevant to your study. If you are not sure if a list item applies to your research, read the appropriate section before selecting a response.

Materials & experimental systems

n/a	Involved in the study	
☐	☒	Antibodies
☐	☒	Eukaryotic cell lines
☒	☐	Palaeontology and archaeology
☐	☒	Animals and other organisms
☒	☐	Clinical data
☒	☐	Dual use research of concern
☒	☐	Plants

Methods

n/a	Involved in the study	
☒	☐	ChIP-seq
☐	☒	Flow cytometry
☒	☐	MRI-based neuroimaging

Antibodies

Antibodies used

Antibodies used are listed in supplementary table two within the manuscript.
Flow Cytometry:
CD19 (1:100) (Clone HIB19, Biolegend, #302208), CD19 (1:200) (Clone HIB19, eBioscience, #48-0199-42), CD20 (1:200) (Clone 2H7, eBioscience, #12-0209-41), CD20 (1:200) (Clone 2H7, eBsiocience, #48-0209-42), CD20 (1:50) (Clone 2H7, Invitrogen, #47-0209-42), CD223 (Lag-3) (1:50) (Clone 11C3C65, Biolegend, #369310), CD223 (Lag-3) (1:50) (Clone 11C3C65, Biolegend, #369308), CD3 (1:100) (Clone UCHT1, Beckman Coulter, #737657), CD3 (1:200) (Clone UCHT1, Life Technologies, #17-0038-42), CD366 (TIM-3) (1:50) (F38-2E2, Biolegend, #345042), CD4 (1:25), (Clone RPA-T4, Life Technologies, #79-0049-42), CD45 (1:50) (Clone J33, Beckman Coulter, #A07784), CD45 (1:50) (Clone 2D1,eBioscience, #48-9459-41), CD45 (1:50) (Clone MEM-28, Thermofisher Scientific, #MA1-10230), CD45 (1:25) (Clone J33, Beckman Coulter, # B36294), CD45RA (1:50) (Clone HI100, BD Pharmingen, #550855), CD62L (1:400) (Clone DREG-56, Biolegend, #304840), CD8 (1:400) (Clone RPA-T8, Biolegend, #301049), CD8 (Clone 1:100, Biolegend, #47-0086-42), CD8 (1:100) (Clone B9.11, Beckman Coulter, #A07756), CD8 (1:100) (Clone OKT-8, eBioscience, #12-0086-42), CD8 (1:200) (Clone OKT-8, eBioscience, #25-0086-42), CD8 (1:50) (Clone OKT-8, eBioscience, #48-0086-42), EGFR (1:2000) (Clone AY13, Biolegend, #352911), EGFR (1:2000) (Clone AY13, Biolegend, #352906), EGFR (1:2000) (Clone AY13, Biolegend, #352904), HLA-DR (1:50) (Clone L243, Exbio, PO-690-T100), IFN-y (1:20) (Clone 45.15, Beckman Coulter, #IM2717U), IL-2 (1:20) (Clone 5344.111, BD Pharmingen, #341116), PD-1 (1:100) (Clone eBioJ105, Invitrogen, #17-2799-42), Streptavidin (1:50) (Life Technologies, #48-4317-82), Streptavidin (1:50) (BD Pharmingen, #554060), Streptavidin (1:50) (Invitrogen, #12-4317-87), Streptavidin (1:50) (eBioscience, #17-4317-82), TNF-a (1:300) (Clone MAb11, eBioscience, #25-7349-82), TIGIT (1:100) (Clone VSTM3, Biolegend, #372708)

Single Cell RNA Seq Hashtag antibodies:
TotalSeq™-A0251 anti-human Hashtag 1 Antibody (GTCAACTCTTTAGCG) (Clone LNH-94; 2M2, Biolegend, 1:100)
TotalSeq™-A0252 anti-human Hashtag 2 Antibody (TGATGGCCTATTGGG) (Clone LNH-94; 2M2, Biolegend, 1:100)
TotalSeq™-A0253 anti-human Hashtag 3 Antibody (TTCCGCCTCTCTTTG) (Clone LNH-94; 2M2, Biolegend, 1:100)
TotalSeq™-B0254 anti-human Hashtag 4 Antibody (AGTAAGTTCAGCGTA) (Clone LNH-94; 2M2, Biolegend, 1:100)
TotalSeq™-B0255 anti-human Hashtag 5 Antibody (AAGTATCGTTTCGCA) (Clone LNH-94; 2M2, Biolegend, 1:100)
TotalSeq™-B0256 anti-human Hashtag 6 Antibody (GGTTGCCAGATGTCA) (Clone LNH-94; 2M2, Biolegend, 1:100)
TotalSeq™-C0258 anti-human Hashtag 8 Antibody (CTCCTCTGCAATTAC) (Clone LNH-94; 2M2, Biolegend, 1:100)

Validation

The antibodies are used for the detection of human proteins in flow cytometry as well as sample specific labelling within single cell RNA sequencing as indicated above. The validation was done by the manufacturers and the results can be shown on their websites. Furthermore, all antibodies were tested before use and optimal dilutions were titrated.

Eukaryotic cell lines

Policy information about cell lines and Sex and Gender in Research

Cell line source(s)	All cell lines were derived from human origin. Raji (Male, Burkitt's Lymphoma), Nalm-6 (Male, ALL), RD114 (Female, Rhabdomyosarcoma), HEK293T (fetus, kidney), Jeko-1 (female, MCL), A549 (male, lung carcinoma), Nur77 Jurkat (male, acute ATL). Nur77-Jurkat were kindly provided by Juno Therapeutics GmbH, a Bristol Myers Squibb company; Raji-ffluc-GFP, Nalm6-ffluc-GFP, RD114, HEK293T cells were kindly provided by the Stanley Riddell laboratory at the Fred Hutchinson Cancer Research Center, Jeko-1 ffluc and A549 ffluc cells were kindly provided by the Michael Hudecek laboratory at the Department of Internal Medicine II, University Hospital Würzburg).
Authentication	No cell line was authenticated. Prior to use, each cell line was validated for surface antigen expression via flow cytometry and assessed for xeno-engraftment in NSG-S mice (take, latency and biodistribution in relevant organs).
Mycoplasma contamination	All cell lines were routinely tested for mycoplasma contamination via PCR and negative.
Commonly misidentified lines (See ICLAC register)	No commonly misidentified cell line was used.

Palaeontology and Archaeology

Specimen provenance	N/A
Specimen deposition	N/A
Dating methods	N/A

☐ Tick this box to confirm that the raw and calibrated dates are available in the paper or in Supplementary Information.

Ethics oversight	N/A

Note that full information on the approval of the study protocol must also be provided in the manuscript.

Animals and other research organisms

Policy information about studies involving animals; ARRIVE guidelines recommended for reporting animal research, and Sex and Gender in Research

Laboratory animals	eight to eleven week old NSG SGM3 (NSG TgCMV-IL3,CSF2,KITLG1Eav/MloySzJ) were purchased from Jackson Laboratories or derived from in house breeding.
Wild animals	This study did not involve wild animals.
Reporting on sex	Both genders were included in mouse experiments.
Field-collected samples	Animals were group-housed under specific pathogen free conditions with constant temperature of 20 °C and ad libitum access to food and water. A 12-hour light/dark cycle was maintained to regulate circadian rhythm with a relative humidity of 50-60%. Following completion of the study, animals were euthanized and not used in further experiments.
Ethics oversight	All animal experiments were approved by the government of Upper Bavaria, Department 5 - Environment, Health and Consumer Protection, ROB-55.2-2532.Vet_02-18-162

Note that full information on the approval of the study protocol must also be provided in the manuscript.

Plants

Seed stocks	N/A
Novel plant genotypes	N/A
Authentication	N/A

Flow Cytometry

Plots

Confirm that:

☒ The axis labels state the marker and fluorochrome used (e.g. CD4-FITC).

☒ The axis scales are clearly visible. Include numbers along axes only for bottom left plot of group (a 'group' is an analysis of identical markers).

☒ All plots are contour plots with outliers or pseudocolor plots.

☒ A numerical value for number of cells or percentage (with statistics) is provided.

Methodology

Sample preparation	Human peripheral blood or cord blood mononuclear cells were isolated vie denstiy gradient centrifugation, washed with PBS and stained for 20 min at 4 °C in darkness. Cell culture cells were washed in FACS buffer and stained accordingly. Red blood cells of murine blood samples, bone marrow and spleen single cell suspensions were lysed with ACT solution before staining.
Instrument	CytoFLEX S, Beckman coulter
Software	FlowJo Software, LLC
Cell population abundance	In each experiment at least 20,000 human hCD45 events were acquired
Gating strategy	Cell culture cells, human or mouse peripheral blood, bone marrow single cell suspensions were serially gated as follows: FSC-H/SSC-H, FSC-Width/SSC-H, following Live/dead discrimation PE/ECD and STII/EGFR or any other relevant combination

☒ Tick this box to confirm that a figure exemplifying the gating strategy is provided in the Supplementary Information.
